# Supplementary material for: Dynamic transcriptomic profiles of zebrafish gills in response to zinc supplementation
Source: BMC Genomics. 2010 Oct 11;11:553. doi: 10.1186/1471-2164-11-553 (PMC3091702; doi:10.1186/1471-2164-11-553)
Supplement: Additional file 2 — Interactive Direct Interaction Network representing the molecular interactions between zinc, copper, iron, calcium and proteins encoded by transcripts changed by zinc supplementation. Mini web-site containing index.html and hyperlinked pages in subdirectory describing a Direct Interaction Network automatically generated based on curated interactions contained within the proprietary PathwayArchitect database. Ovals represent proteins and the circles symbolize metal ions. Objects are coloured by their abundance in zebrafish at the time-point they were significantly different from the control is a scale from -4 fold (dark green) to +4 fold (dark red). Where significant differences were found at more than one time-point, the colour overlay shows expression at the first instance. Dark blue squares denote 'binding', and light blue squares 'expression'; green squares stand for 'regulation', green diamonds for 'metabolism', and green circles for 'promoter binding'. Arrow heads indicate directionality of the interaction where annotated. All nodes and edges can be further interrogated by selecting the relative area of the image. [file 1471-2164-11-553-S2.zip › PathwayArchitect Zn xs DIN/440505.html]

# REGULATION:

|  |  |
| --- | --- |
| Type | REGULATION |
| Effect | Positive |


---

|  |  |
| --- | --- |
| Score | 0 |


---

|  |  |
| --- | --- |
| Reference Count | 7 |


---

|  |  |
| --- | --- |
| Mechanism | Unknown |


---

|  |  |
| --- | --- |
| Reference:0 || Sentence | "The binding of Ins(1,4,5)P3 to Ins(1,4,5)P3R1 and Ins(1,4,5)P3R3 was differentially regulated by Ca2+." |
| PMID | 9065781 |
| Year | 1997 |
| Species | Human |
| Journal | Biochem J |
| RefScore | 1 |
| Source | PArchNLP |
  |
|  |  |


---

|  |  |
| --- | --- |
 Reference:1 || Sentence | "These results indicate that (1) both Ins(1,4,5)P3R1 and Ins(1,4,5)P3R3 have at least two affinity states, (2) Ca2+ regulates interconversions between these states, and (3) Ca2+ regulates the binding of Ins(1,4,5)P3 to Ins(1,4,5)P3R1 and Ins(1,4,5)P3R3 in opposite manners." |
| PMID | 9065781 |
| Year | 1997 |
| Species | Human |
| Journal | Biochem J |
| RefScore | 1 |
| Source | PArchNLP |
  ||  |  |


---

|  |  |
| --- | --- |
 Reference:2 || Sentence | "In this study, we have, therefore, investigated the effects of Ca2+ and Mg2+ on Ins(1,4,5)P3 binding to membranes of the myeloid cell line HL-60 and assayed in parallel phospholipase C activity and Ins(1,4,5)P3 degradation." |
| PMID | 8389758 |
| Year | 1993 |
| Species | Human |
| Journal | J Biol Chem |
| RefScore | 2 |
| Source | PArchNLP |
  ||  |  |


---

|  |  |
| --- | --- |
 Reference:3 || Sentence | "The effects of Ca2+ and Mg2+ on Ins(1,4,5)P3 binding were conserved after solubilization of HL-60 membranes." |
| PMID | 8389758 |
| Year | 1993 |
| Species | Human |
| Journal | J Biol Chem |
| RefScore | 0 |
| Source | PArchNLP |
  ||  |  |


---

|  |  |
| --- | --- |
 Reference:4 || Sentence | "However, the sensitivity of the Ca2+ release mechanism to Ins(1,4,5)P3 is highly dependent on experimental conditions and on membrane potential. 4." |
| PMID | 8013729 |
| Year | 1994 |
| Species | Human |
| Journal | Int J Biochem |
| RefScore | 1 |
| Source | PArchNLP |
  ||  |  |


---

|  |  |
| --- | --- |
 Reference:5 || Sentence | "Thus both Tg and tBuBHQ release Ca2+ from a pool that totally overlaps the Ins(1,4,5)P3-sensitive pool without affecting Ins(1,4,5)P3 function." |
| PMID | 8363562 |
| Year | 1993 |
| Species | Human |
| Journal | Biochem J |
| RefScore | 0 |
| Source | PArchNLP |
  ||  |  |


---

|  |  |
| --- | --- |
 Reference:6 || Sentence | "The augmentation of Ins(1,4,5)P3-mediated currents by CIF was bell-shaped with regard to Ins(1,4,5)P3 concentration, reminiscent of the regulatory influence of Ca2+ on Ins(1,4,5)P3 responses." |
| PMID | 8809059 |
| Year | 1996 |
| Species | Human |
| Journal | Biochem J |
| RefScore | 1 |
| Source | PArchNLP |
  |


---

|  |  |
| --- | --- |
